# Biflavonoid-Induced Disruption of Hydrogen Bonds Leads to Amyloid-β Disaggregation

**DOI:** 10.3390/ijms22062888

**Published:** 2021-03-12

**Authors:** Peter K. Windsor, Stephen P. Plassmeyer, Dominic S. Mattock, Jonathan C. Bradfield, Erika Y. Choi, Bill R. Miller, Byung Hee Han

**Affiliations:** 1 Department of Chemistry, Truman State University, Kirksville, MO 63501, USA; pkw4146@truman.edu (P.K.W.); stephenplassmeyer@gmail.com (S.P.P.); dsm7762@truman.edu (D.S.M.); jcb6582@truman.edu (J.C.B.); 2 Department of Pharmacology, A.T. Still University, Kirksville, MO 63501, USA; erikachoi92@gmail.com

**Keywords:** neurodegenerative disease, Alzheimer’s disease, amyloid aggregation, anti-amyloid compounds, drug discovery, naturally occurring flavonoids, polyphenols, amentoflavone

## Abstract

Deposition of amyloid β (Aβ) fibrils in the brain is a key pathologic hallmark of Alzheimer’s disease. A class of polyphenolic biflavonoids is known to have anti-amyloidogenic effects by inhibiting aggregation of Aβ and promoting disaggregation of Aβ fibrils. In the present study, we further sought to investigate the structural basis of the Aβ disaggregating activity of biflavonoids and their interactions at the atomic level. A thioflavin T (ThT) fluorescence assay revealed that amentoflavone-type biflavonoids promote disaggregation of Aβ fibrils with varying potency due to specific structural differences. The computational analysis herein provides the first atomistic details for the mechanism of Aβ disaggregation by biflavonoids. Molecular docking analysis showed that biflavonoids preferentially bind to the aromatic-rich, partially ordered N-termini of Aβ fibril via the π–π interactions. Moreover, docking scores correlate well with the ThT EC_50_ values. Molecular dynamic simulations revealed that biflavonoids decrease the content of β-sheet in Aβ fibril in a structure-dependent manner. Hydrogen bond analysis further supported that the substitution of hydroxyl groups capable of hydrogen bond formation at two positions on the biflavonoid scaffold leads to significantly disaggregation of Aβ fibrils. Taken together, our data indicate that biflavonoids promote disaggregation of Aβ fibrils due to their ability to disrupt the fibril structure, suggesting biflavonoids as a lead class of compounds to develop a therapeutic agent for Alzheimer’s disease.

## 1. Introduction

Alzheimer’s disease (AD) remains the leading cause of dementia worldwide, with incidence rates expected to rise steadily in the coming decades [1]. Presently available pharmacological treatments help to manage symptoms by compensating for dysregulation of cholinergic and glutamatergic signaling but with minimal effect to either slow or halt the disease process [2]. Towards defining a fundamental molecular etiology of AD as a target for drug development, the amyloid cascade hypothesis has been a leading model for linking the deposition of amyloid β (Aβ) peptide with the onset of neurodegeneration and cognitive decline [3]. Generated from the aberrant cleavage of amyloid precursor protein (APP), Aβ peptide accumulates through a process of misfolding and aggregation in which natively α-helical monomers associate to form an ensemble of oligomeric intermediates. These intermediates in turn promote the formation of insoluble β-rich fibrils [4]. Although a precise mechanism for aggregation remains uncertain, fibrils display self-templating growth and serve as nucleation sites for further accelerated aggregation [5]. Diffusible oligomeric species, either generated as intermediates of this aggregation pathway or derived from fragmentation of extant fibrils [6], have been shown to induce neurotoxic stress by disrupting cell membrane integrity, signal transduction, and other key homeostatic mechanisms [7].

Given the centrality of Aβ aggregates to the disease process, perturbation of the aggregation pathway has become a popular target for developing novel pharmacological interventions [8]. Previous studies have focused on the discovery and design of small molecules [9], peptides [10], peptidomimetics [11] which help prevent aggregates from forming or increase their solubility and clearance from the brain. Numerous natural products have been investigated in AD models for their neuroprotective effects, which are mediated in part by direct interactions with Aβ fibrils and oligomers [12]. Flavonoids are one such class of naturally occurring polyphenols with well characterized neuroprotective effects in the presence of Aβ and other stressors [13]. Structurally diverse, these compounds are generally categorized as mono- or biflavonoids depending on the presence of either one or two functional flavone units. Similar to other polyphenolic aggregation inhibitors [14], there is evidence that flavonoids promote the accumulation of less toxic, off-target oligomers and thereby reduce amyloid load [15]. However, compared to monoflavonoids, bioflavonoids more potently and specifically reduce amyloid-associated toxicity [16].

The precise mechanism of small molecular aggregation inhibitors remains poorly understood [17], largely due to the structural heterogeneity and complex aggregation dynamics of Aβ. Monomeric and oligomeric intermediates are metastable under physiological conditions, and the aggregates formed display a high degree of polymorphism. Furthermore, several isoforms of Aβ naturally occur, with Aβ_40_ and Aβ_42_ being the most abundant, and each isoform has distinct aggregation kinetics and structural features [18]. Salt-bridge formation between the side chain of K28 and the carboxylate of the additional C-terminal A42 in Aβ_42_ modifies the misfolding pathway relative to Aβ_40_ [19], contributing to faster aggregation rates and aggregates with greater neurotoxicity [20]. Methods for selecting monomorphic fibrils by seeding and careful selection of conditions have allowed for reproducible control over the fibril species generated in vitro, allowing for structural determination via electron microscopy [21] as well as both solution and solid-state NMR-based structural modeling [22]. Structural information of inhibitor interactions with monomers in solution has been inferred through NMR experiments [23]; however, such structural information regarding oligomers and fibrils remains experimentally inaccessible [24].

Given the limitations of experimental methods, computational modelling has emerged as a valuable tool for studying Aβ-inhibitor interactions in atomistic detail [25]. Through the use of molecular docking and molecular dynamics (MD) simulations, detailed models have been developed to describe how small molecule inhibitors bind to key structural motifs of numerous Aβ polymorphs [26]. With advantages in speed and throughput, molecular docking provides a powerful tool for screening leads and interrogating potential binding sites on Aβ [27]. Docked poses can be further refined using unrestrained MD simulations which also reveal key conformational changes and mechanistic details underlying recognition and binding specificity. Ultimately, this modeling strategy can illustrate how inhibitor-induced structural changes lead to fibril destabilization and disassembly.

There are growing lines of evidence that biflavonoids such as amentoflavone afford the neuroprotective effects on many in vitro as well as in vivo settings of neurological diseases, including strokes [28,29], Alzheimer’s disease [30,31,32,33], and Parkinson’s disease [34]. Recently, we and others have reported that biflavonoids not only inhibit the formation of Aβ_40_ and Aβ_42_ fibrils but also disaggregate preformed-Aβ fibrils with varying efficacy [35,36]. For example, we found that amentoflavone most potently inhibits fibrillization of Aβ_42_ and disaggregates Aβ_42_ fibrils in a cell-free assay system utilizing thioflavin T (ThT). A structure-activity relationship exhibited that the substitution of hydroxyl groups with apolar functional groups attenuates the effects of biflavonoids on Aβ_42_ disaggregation, suggesting the importance of hydrogen bond donors at key positions. However, the structural basis of anti-amyloidogenic activity of biflavonoids and the interactions between Aβ_42_ and biflavonoids at the atomic levels remained to be investigated.

**Figure 1:**
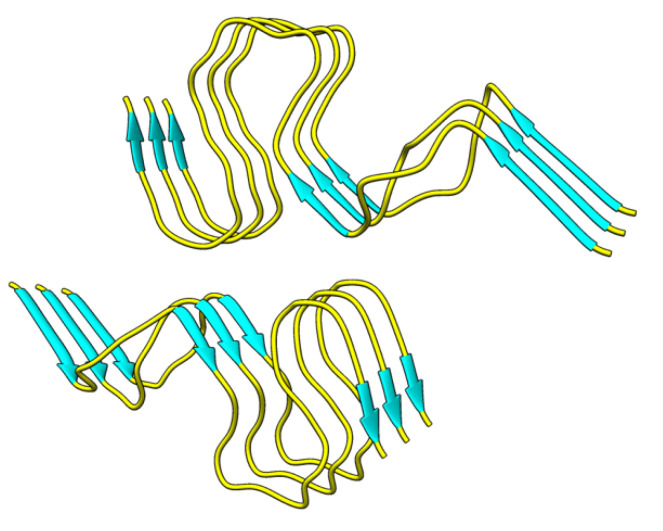
Disease-relevant polymorph of Aβ_42_ fibril (PDB: 2NAO) used for molecular docking and molecular dynamic (MD) simulations. β-structured regions are shown in cyan and non-β-structured regions are shown in yellow.

In the present study, we chose five amentoflavone-type biflavonoids to examine the structure–activity relationship for the Aβ_42_ disaggregating activity by the ThT fluorescence assay method. Although biflavonoids have been studied previously, we utilized computational methods to provide the first extensive structural description of how these biflavonoids promote disaggregation of Aβ_42_ fibrils. To further determine how minor structural variations in each position of hydroxyl groups contribute to observed differences in inhibitor activity on an atomic level, molecular modeling and molecular dynamic simulations were employed. We used an all-atom structure for a disease-relevant polymorph of Aβ_42_ fibril [37] to model the site-specific binding and disaggregation mechanisms of several biflavonoids (Figure 1). The model structure exhibits peptides with an S-shaped fold forming dimeric layers with C_2_ symmetry and has been characterized in independent studies of Aβ_42_ fibrils [27,37]. The structure includes all 42 residues, including the partially ordered N-terminal residues, allowing for a complete evaluation of potential binding sites. Using the entire fibril surface as a search space, molecular docking simulations were performed to identify optimal binding poses for five biflavonoids. Conformational perturbations to the fibril in complex with these poses were further examined through explicit-solvent unrestrained MD simulations on the microsecond timescale. Analysis of these trajectories highlights specific binding interactions near the N-terminus of the Aβ peptides in the fibril model, and large-scale structural changes occurring on the nanosecond timescale suggest a mechanism for biflavonoid-driven structural remodeling.

## 2. Results and Discussion

### 2.1. Thioflavin T Fluorescence Assay

**Figure 2:**
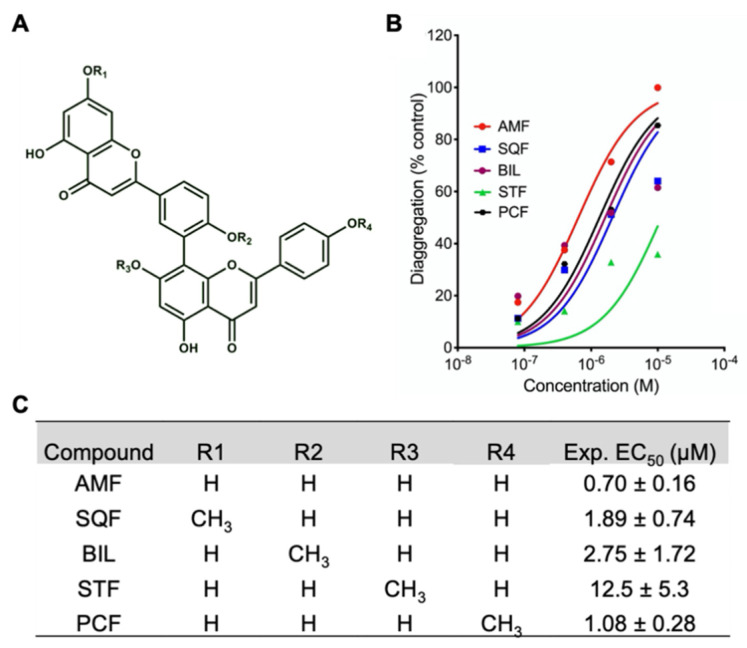
**Thioflavin (**ThT) assay disaggregation results from five biflavonoids. (**A**) Generic biflavonoid structure. R-groups for the different biflavonoids investigated in this study can be found in (**C**). AMF: amentoflavone; SQF: sequoiaflavone; BIL: bilobetin; STF: sotetsuflavone; PCF: podocarpusflavone. (**B**) Disaggregating activity of biflavonoids on pre-formed Aβ_42_ fibrils. Aβ_42_ fibrils (20 μM) were incubated with ThT (5 μM) in the presence of various concentrations of test compounds. Percent changes in fluorescent intensity of ThT vs. concentrations of the compounds were plotted. (**C**) The half-maximal effective concentration (EC_50_) values of the test compounds on disaggregation of Aβ fibrils were calculated and presented as mean ± S.E.M.

The highly aromatic biflavonoid structure can be seen in Figure 2. The R-groups represent either a methyl group or a hydrogen atom, resulting in a methoxy substituent or hydroxyl substituent, respectively. In order to determine the role of hydrogen bonding functional groups, a series of biflavonoids was chosen with methoxy groups substituted at key positions on the amentoflavone (AMF) scaffold. The different R-groups for the biflavonoids used in this study can be found along with corresponding experimentally derived EC_50_ values determined from the Thioflavin T fluorescence assays (Figure 2). The assay results reveal that AMF exhibits the most potent effect on promoting disaggregation of Aβ_42_ fibrils while STF is the least effective. Sirimangkalakitti et al. recently published results testing the inhibitory effects of these biflavonoids with Aβ_40_ and also found that STF had a lower inhibitory effect relative to other the mono-methoxy bioflavonoids [35]. They present that AMF, SQF, BIL, and PCF all had similar inhibitory effects, whereas we found that these biflavonoids differed in their effects. We believe these discrepancies are due to the structural differences between Aβ_40_ and Aβ_42_.

In line with results from the ThT assay (Figure 2), we have recently reported that AMF directly disrupted fibrillar structure of Aβ_42_, resulting in the conversion of β-sheet Aβ fibrils to amorphic aggregates as determined by atomic force microscopy [38]. The experimental results from the thioflavin T fluorescence assays demonstrate that biflavonoid compounds exhibit different activities on Aβ fibril disaggregation. Methoxy groups at positions R_1_ and R_4_ have lower penalties on potency relative to methoxy groups at R_2_ and R_3_ which exhibit larger penalties on potency. Nevertheless, these results do not suggest atomistic details as to how these biflavonoids destabilize the Aβ fibril. Therefore, molecular docking and molecular dynamics (MD) were employed to elucidate how the biflavonoids affect Aβ fibril stability.

### 2.2. Molecular Docking of Biflavonoids

**Figure 3:**
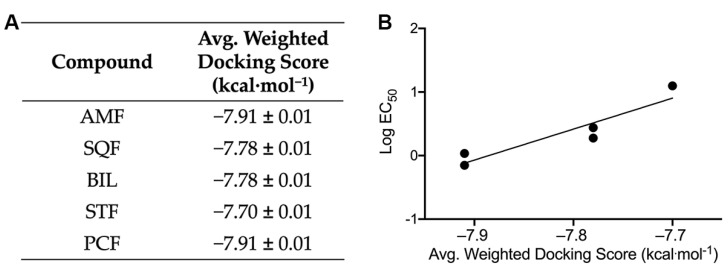
Results from docking biflavonoids to Aβ_42_ (PDB: 2NAO). (**A**) Average weighted docking scores ± S.E.M. for the most negative binding cluster for each biflavonoid calculated from the AutoDock Vina scoring function via the PyRx interface. Simulations were performed with Aβ_42_ in the presence of amentoflavone (AMF), sequoiaflavone (SQF), bilobetin (BIL), sotetsuflavone (STF), and podocarpusflavone (PCF). (**B**) Experimental EC_50_ values plotted against the average weighted AutoDock Vina docking scores for each biflavonoid cluster bound to the Aβ fibril showing a positive correlation (R^2^: 0.866, *p* = 0.022).

Molecular docking was carried out using the AutoDock Vina scoring function via the PyRx interface. An exhaustive docking method was used to generate sufficient sampling to be compared to EC_50_ values. The docking results for the most negative binding cluster for each biflavonoid can be found in Figure 3A. Furthermore, there is a significant positive correlation between the experimental EC_50_ values from the ThT fluorescence assays and the docking scores for the biflavonoids (Figure 3B).

**Figure 4:**
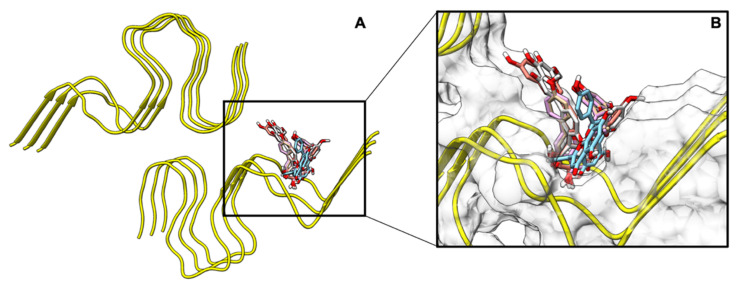
Depiction of the top docking poses from PyRx. (**A**) Aβ_42_ fibril structure (PDB: 2NAO) used for molecular docking and molecular dynamics (MD). (**B**) The most negative binding conformation from the docking calculations for the five biflavonoids overlaid in the N-terminal pocket of the Aβ_42_ fibril.

Additionally, the most negative binding cluster for each biflavonoid was located near the partially ordered N-terminal residues 6–14 of the Aβ fibril (Figure 4). The preferential binding of each molecule to the N-terminal pocket was likely due to the presence of aromatic residues (H6, Y10, H13), and the ability of the molecule to fill the space in the pocket. The highly aromatic biflavonoid formed π–π interactions with the aromatic-rich N-terminal pocket of the fibril to stabilize the biflavonoid-fibril complex. A previous computational study has suggested that small aromatic molecules like biflavonoids primarily interact with C-terminal residues and secondarily interact with N-terminal residues [39]. Discrepancies in the observed primary binding site are most likely due to differences in the Aβ polymorph used in each study. Wang et al. used a pentameric Aβ fibril that was modeled after an incomplete NMR-derived Aβ fibril. The resulting fibril did not contain the N-terminal pocket that is present in the disease-relevant polymorph used in the current study. Recently, a study by Sun et al. [36] also completed docking experiments which confirmed that AMF preferentially interacts with the N-terminal of the Aβ fibril. While Sun et al. observed similar interactions between the Aβ fibril and amentoflavone, the study presented here used a disease-relevant polymorph of the Aβ fibril (PDB: 2NAO) as opposed to other Aβ_40_ and Aβ_42_ structures. Here, a rigorous docking method was used to confirm biflavonoid binding modes and docking scores. Furthermore, the present study docked multiple biflavonoids, confirming that the proposed binding pocket is consistent with biflavonoid variation.

### 2.3. Molecular Dynamics

In addition to molecular docking, explicit-solvent unrestrained MD was performed to study the dynamic interactions between the Aβ fibril and biflavonoid molecules. The Aβ fibril complexed to the most negative binding mode for each biflavonoid from our initial docking screen was used as the starting structure for each simulation. Additionally, a biflavonoid free simulation was performed to evaluate the behavior of the Aβ fibril in the absence of a biflavonoid. Each system was minimized, heated, and equilibrated prior to unrestrained MD in an effort to minimize the bad contacts of the system. Each system was then allowed to propagate for 1.0 μs. Various analyses were performed to analyze the collected data, including root mean square deviation (RMSD), per-residue free energy decomposition analysis, principal component analysis (PCA), secondary structure analysis, and hydrogen bond analysis.

#### 2.3.1. Structural Dynamics

**Figure 5:**
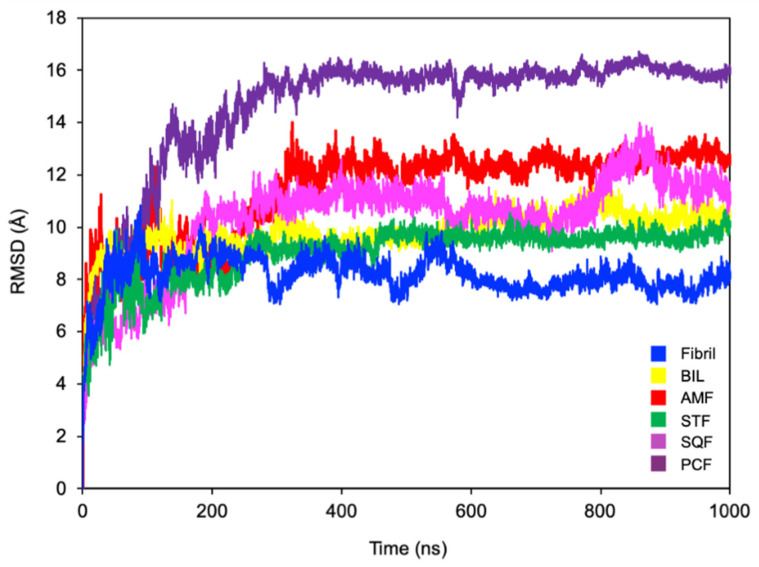
Comparative all atom RMSD for ligand-free and ligand-bound simulations using the equilibrated structure for each simulation as the reference structure. Simulations were performed with Aβ_42_ alone (Fibril) or in the presence of bilobetin (BIL), amentoflavone (AMF), sotetsuflavone (STF), sequoiaflavone (SQF), and podocarpusflavone (PCF).

RMSD can be used as a measure of conformational stability over the course of a simulation. Figure 5 shows the all-atom RMSD for the ligand-free and ligand-bound simulations over 1000 ns. The equilibrated structure for each simulation was used as the reference structure for these calculations. It is clear from Figure 5 that the ligand-free simulation exhibits the smallest deviation from the starting structure. In contrast, the ligand-bound simulation with PCF exhibits the largest deviation from the starting structure. All of the ligand-bound simulations exhibit a larger deviation from the starting structure relative to the ligand-free simulation, indicating that the biflavonoids cause conformational changes within the fibril, promoting disaggregation. Discrepancies between EC_50_ values and RMSD are likely a result of the smaller timescales sampled in our simulations (1.0 μs) relative to in vitro disaggregation (hours). While full disaggregation is able to be observed in vitro, our simulations only represent a small portion of this disaggregation process. Additionally, EC_50_ values were determined using full Aβ aggregates, whereas simulations were carried out using a six-peptide aggregate.

#### 2.3.2. Per-Residue Free Energy Decomposition Analysis

**Figure 6:**
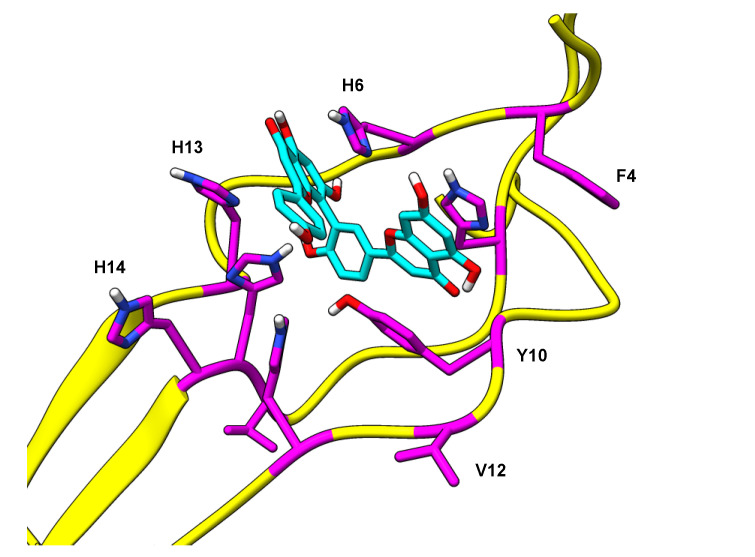
Amentoflavone in the N-terminal pocket of the Aβ_42_ fibril being stabilized by F4, H6, Y10, V12, H13, and H14. This image is representative of the π-π interactions that were observed in the other biflavonoid simulations.

Solvation free energy for the biflavonoid-fibril complexes, ΔG_decomp_, was measured and decomposed per-residue to characterize the interactions stabilizing each inhibitor within the binding pocket. The major residues contributing to complex stability were F4, H6, Y10, V12, H13, and H14 (Table 1). During the simulations, these non-polar and/or aromatic residues adjust such that they form favorable van der Waals (VDW) interactions. For these residues, comparison of the VDW, electrostatic, and solvation energy contributions to the total solvation free energy shows that the average ΔG_vdw_, ΔG_ele_, and ΔG_nonpolar_ values are stabilizing (negative) whereas the average ΔG_polar_ values are destabilizing (positive). Favorable π-π interactions formed with aromatic residues F4, H6, Y10, H13, and H14 (Figure 6), as estimated by ΔG_vdw_, account for the greatest stabilizing component to ΔG_decomp_. Full ΔG_decomp_ data can be found in the Supporting Information. Combining the most stable conformations from docking and the free energy analysis from MD, we hypothesize that the favorable biflavonoid–fibril π–π interactions promote initial binding of the biflavonoid to the Aβ fibril and help maintain the overall protein-ligand complex.

#### 2.3.3. Principal Component Analysis

**Figure 7:**
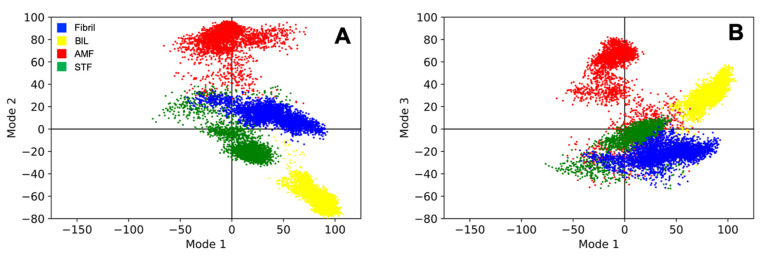
Principal component analysis (PCA) results from the ligand-free simulation and three representative ligand-bound simulations. Projection of the individual trajectories onto the first and second dominant modes of motion and (**A**) first and third dominant modes of motion (**B**) for three ligand-bound simulations and the ligand-free simulation showing the difference in motion between systems. Simulations were performed with Aβ_42_ alone (Fibril) or in the presence of bilobetin (BIL), amentoflavone (AMF), and sotetsuflavone (STF). Projections for the second and third dominant modes of motion can be found in the Supporting Information along with the data for all six simulations.

Principal component analysis (PCA) was used to reduce the dimensionality of the MD simulations to elicit the dominant modes of motion for the Aβ fibril–biflavonoid complex [40]. This was in an effort to elucidate the motions and conformations that characterize the disaggregation of the Aβ fibril. The trajectories of the five ligand-bound simulations and the ligand-free simulation were concatenated into a single conformational ensemble, and five dominant modes of motion were extracted using PCA [41]. The equilibrated fibril structure was used as the reference structure for each system. The trajectories were stripped of all atoms except the ⍺-carbons of the peptide backbone to isolate the motion of the peptides [42]. Figure 7A,B show PCA scatter plots generated for three ligand-bound simulations and the one ligand-free simulation projected on to the first and second dominant modes of motion and first and third dominant modes of motion. Data for the second and third dominant modes of motion can be found in the Supporting Information along with the PCA data for all six simulations. The scatter plot shows that the eigenvalues from the MD trajectory are varied between the four systems. This suggests that the disaggregation mechanism is unique between biflavonoids even though PCA is unable to reveal how these mechanisms are structurally different. The ligand-bound simulation trajectories show less variation in the dominant normal mode compared to the ligand-free simulation indicating that the ligand-bound simulations are less dynamic than the ligand-bound simulations. The ligand-bound complexes move further away from the starting conformations and stabilize after reaching the new conformation, reflected by denser clusters for PCA but increased RMSD. This can be visually observed in the MD trajectories of the ligand-bound simulations as the biflavonoids collapse the fibril structure thus limiting the degrees of motion available to the complex.

Using the Normal Mode Wizard plug-in of VMD, these modes of motion were visualized in the form of porcupine plots. The porcupine plots for the first, second, and third dominant modes of motion can be found in the Supporting Information, where the vectors represent the general direction of the ⍺-carbon for each amino acid in the fibril. The N-termini and core of each set of peptides fold towards each other while the C-termini of each set also fold towards each other but in the opposite direction relative to the N-termini and core. The second and third dominant modes of motion show similar motions. The observed folding motion twists the Aβ peptides within the fibril and decreases the hydrogen bonding potential in the peptide backbone. This motion destabilizes the β-sheets within the fibril, promoting collapse of the fibril structure.

#### 2.3.4. Secondary Structure Analysis

The rigid structure and stability of the Aβ_42_ fibril has been attributed to high β-sheet content throughout the fibril. The total β-sheet content of the fibril was quantified over the course of the simulation to observe whether the biflavonoids disrupted the secondary structure. Specifically, the secstruct module of cpptraj was used to assign a DSSP secondary structure label to each residue for each frame of a trajectory. The percentage of residues designated as parallel β-strand was reported as the β-sheet content.

**Figure 8:**
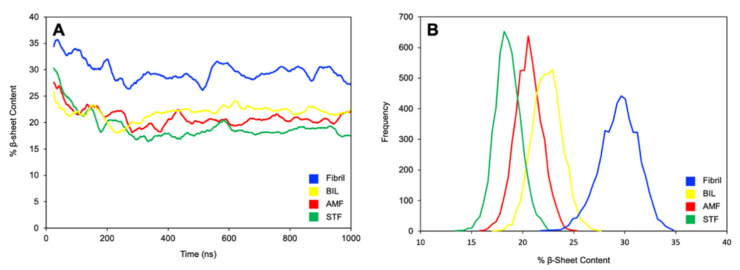
Results from secondary structure analysis on the molecular dynamic (MD) simulations. Simulations were performed with Aβ_42_ alone (Fibril) or in the presence of bilobetin (BIL), amentoflavone (AMF), and sotetsuflavone (STF). (**A**) Change in % β-sheet content over time for the MD trajectories averaged every 25 ns. Ligand-bound simulation shows greater β-sheet content relative to the ligand-free simulation. Note that all simulations started at the same % β-sheet content (~35%), but due to the moving average calculation, the first 25 ns were not able to be captured graphically resulting in apparent varying starting points. (**B**) Histogram showing the β-sheet conformations sampled over the last 500 ns of the MD trajectories. The ligand-free simulation shows greater β-sheet content relative to the ligand-bound simulations. For visual clarity, we have only included the data for four simulations here; secondary structure data for all simulations can be found in the Supporting Information.

All simulations started with the fibril at 35% β-sheet content and decreased steadily over the first 500 ns. The decrease in β-sheet content stabilized during the last 500 ns for each simulation. The change in β-sheet content over time for the ligand-bound simulations and ligand-free simulation can be seen in Figure 8A. The ligand-free simulation showed a slight decrease in content relative to the starting structure, stabilizing at 29% following equilibration and relaxation. The ligand-bound simulations showed a larger decrease in β-sheet content relative to the ligand-free simulation, stabilizing around 20%. This larger decrease in β-sheet content suggests that the presence of biflavonoids promotes new structural conformations of the Aβ_42_ fibril.

The last 500 ns of the ligand-bound simulations were averaged and compared in order to quantify the efficacy and differentiate the biflavonoids. The histogram in Figure 8B depicts the frequency of the β-sheet content of the conformations sampled. Full data for all six simulations can be found in the Supporting Information. The large difference in conformations between the ligand-free simulation and ligand-bound simulation confirms that the presence of the biflavonoid induces a conformational change in the fibril structure. Given the large overlap between the conformations sampled for the ligand-bound simulations, it is concluded that the efficacy of biflavonoids cannot be differentiated solely using β-sheet content on this time scale. However, it is believed that the decrease in β-sheet content is a step towards disaggregation. The relatively short time scale of these simulations is also likely an explanation for the lack of direct correlation between the measured β-sheet content with the experimentally measured EC_50_ values from the ThT fluorescence assays.

#### 2.3.5. Hydrogen Bond Analysis

**Figure 9:**
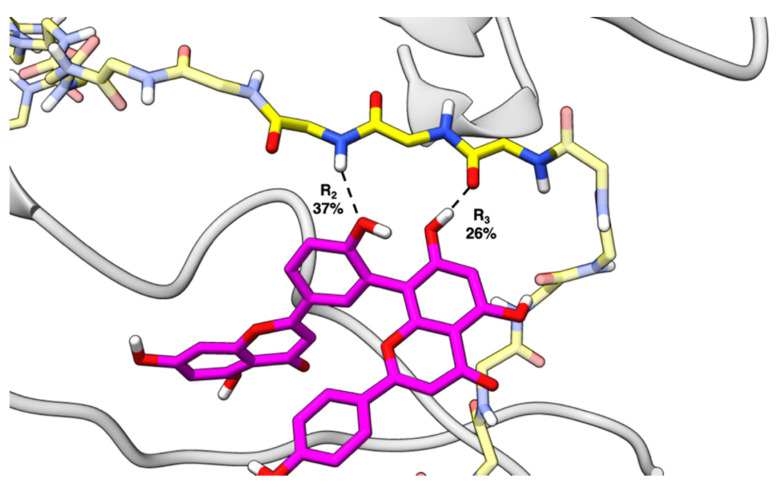
Amentoflavone (AMF) forming hydrogen bonds between R_2_ and R_3_ and the peptide backbone with hydrogen bond formation percentages by respective R-group (amino acid R-groups are stripped for clarity).

Hydrogen bond analysis was used to investigate hydrogen bonding interactions that were important for biflavonoid-fibril binding. Specifically, the hydrogen bonding between the biflavonoid R-groups and the fibril were investigated to determine how R-group differentiation affected interactions with the fibril. Biflavonoids with hydroxyl groups at R_2_ or R_3_ formed hydrogen bonds with the peptide backbone over a significant portion of the simulation. Hydrogen bond formation between hydroxyl groups at R_2_/R_3_ and the peptide backbone suggests a reason for β-sheet destabilization (Figure 9). The hydrogen bonding between hydroxyl R_2_/R_3_ and the peptide backbone displaces the peptide backbone to peptide backbone hydrogen bonding that is responsible for β-sheet formation. Additionally, the presence of hydroxyl groups at R_2_/R_3_ and their hydrogen bonding activity is consistent with the experimental EC_50_ values. When R_2_/R_3_ are both hydroxyl groups (e.g., AMF, SQF, and PCF), significant hydrogen bonding is observed with the peptide backbone, and this is consistent with the lower EC_50_ values. When either R_2_ or R_3_ are occupied by methoxy groups (e.g., BIL and STF), hydrogen bonding with the peptide backbone does not occur which is consistent with the higher EC_50_ values. This further supports that hydroxyl groups at R_2_/R_3_ are important for fibril destabilization. No consistent hydrogen bonding trends were observed between R_1_/R_4_ and the peptide backbone. R_1_/R_4_ mainly formed hydrogen bonds with amino acid side chains and solvent and do not appear to contribute significantly to fibril destabilization. The percentage of frames with hydrogen bonds between the peptide backbone and R_2_/R_3_ for each biflavonoid can be found in Table 2.

**Figure 10:**
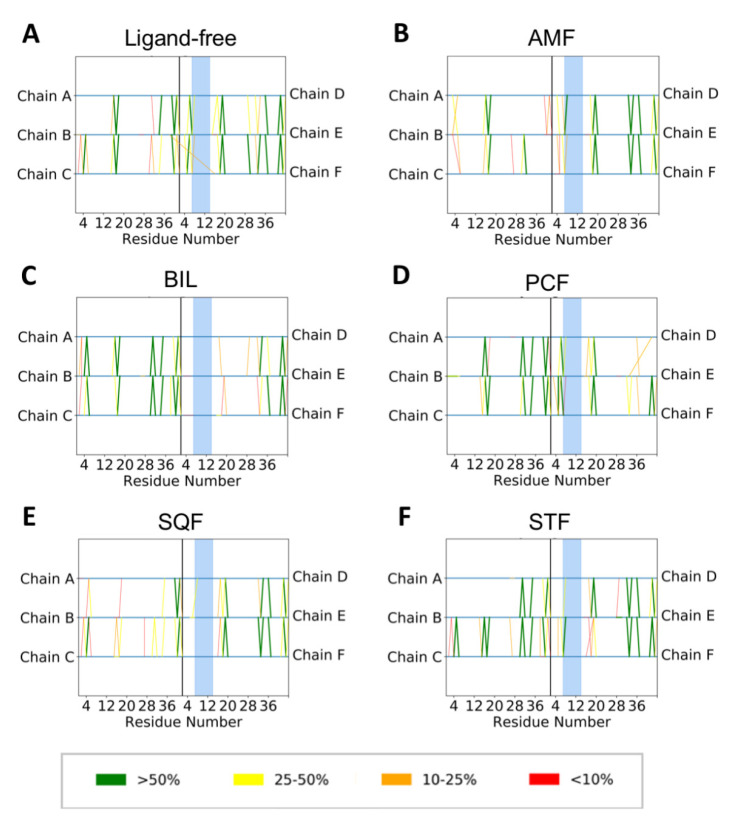
Protein backbone hydrogen bonding analysis derived from simulations of Aβ_42_ with (**A**) no ligand, (**B**) amentoflavone (AMF), (**C**) bilobetin (BIL), (**D**) podocarpusflavone (PCF), (**E**) sequoiaflavone (SQF), and (**F**) sotetsuflavone (STF). Hydrogen bonds percentages were calculated over the each 1.0 μs simulation, and were categorized by the percent of frames with backbone hydrogen bonds present: >50% (green), 25–50% (yellow), 10–25% (orange) and less than 10% (red). Each line depicted shows a backbone hydrogen bond present during the simulation to and from one of the six peptides (Chains **A**–**F**) from the original structure (PDB 2NAO). The blue highlighted region depicts the area with the most direct contact with each ligand from docking. Larger images of each panel with amino acids 1–42 listed explicitly can be found in the SI.

Hydrogen bonding analysis was also performed to investigate the backbone hydrogen bonding that stabilizes the Aβ fibrils in the absence and presence of the biflavonoid inhibitors. The original NMR structure (PDB: 2NAO) contains six peptides (Chains A–F) where Chains A–C forms one β-sheet and Chains D–F form another sheet (Figure 1). We observed a decrease in total backbone hydrogen bonding for all simulations with a biflavonoid bound to the fibril relative to the ligand-free simulation (Table S2). Throughout the ligand-free simulation, more backbone hydrogen bonds were present for longer in Chains D–F than Chains A–C (Figure 10A). The docking results demonstrate that the biflavonoid ligands preferentially bound to Chains D–F in all instances, specifically near residues 6–14 with multiple aromatic amino acids. In all ligand-bound simulations (Figure 10B–F), we observed a noticeable decrease in prevalent backbone hydrogen bonding (>50%) for Chains D–F in the regions where the biflavonoids were bound. The decrease was most significant at or near where the biflavonoids interacted most with the fibril (blue shaded region of Figure 10B–F). These results further demonstrate that the loss of β-sheet character observed during ligand-bound simulations (Figure 8) arises from the loss of backbone hydrogen bonding between the Aβ_42_ peptides. This suggests that the presence of the biflavonoids directly disrupts the backbone hydrogen bonds that stabilize the secondary structure of the Aβ fibril.

## 3. Materials and Methods

### 3.1. Thioflavin T Fluorescence Assay

To test the effects of biflavonoids on promoting disaggregation of Aβ_42_ fibrils, we performed the ThT fluorescent assay per the published method [38,43,44]. Briefly, monomeric Aβ_42_ (American Peptide Company, Sunnyvale, CA, USA) was dissolved in hexafluoroisopropanol, air dried, and dissolved in dimethyl sulfoxide (DMSO) and kept at −20 °C until use. Aβ_42_ was then diluted in PBS buffer (50 mM NaH_2_PO_4_ and 100 mM NaCl, pH 7.40) and added to a 96-well plate containing final concentrations of 20 μM Aβ_42_, 5 μM ThT, and incubated at 37 °C for 24 h to generated Aβ_42_ fibrils. Amentoflavone (AMF) and bilobetin (BIL) were kindly gifted from Dr. Sam Sik Kang at Seoul National University, Seoul, South Korea. Sequoiaflavone (SQF), sotetsuflavone (STF), and podocarpusflavone (PCF) were provided by Dr. Kiyotaka Koyama at Meiji Pharmaceutical University. To determine the ability of the biflavonoids on promoting disaggregation of Aβ_42_ fibrils, the test compounds at various concentrations were added to the preformed Aβ_42_ fibrils and continued incubating at 37 °C for 6 h. The ThT fluorescence was measured at 508 nm (excitation at 460 nm) on a Bio-Tek plate reader. The half maximum effective concentration (EC_50_) values were calculated using the GraphPad Prism 8 software (San Diego, CA, USA).

### 3.2. Docking

Structures for five biflavonoids—amentoflavone (AMF), bilobetin (BIL), podocarpusflavone (PCF), sequoiaflavone (SQF), and sotetsuflavone (STF)—were built in the Avogadro molecule editor and viewer [45] and parameterized using Gaussian09 (Wallingford, CT, USA) [46] at the Hartree-Fock/6-31G* level of theory. To identify potential inhibitor binding sites, molecular docking calculations were performed with AutoDock Vina (La Jolla, CA, USA) [47] via the PyRx interface. The parametrized structures were docked to a receptor model of a disease-relevant Aβ_42_ fibril obtained from the Protein Databank (PDB: 2NAO) [37]. To generate an initial MD structure, each biflavonoid was docked to the fibril structure using the entire fibril surface as the search space. Eight poses were generated per biflavonoid and ranked by docking score. For each biflavonoid, the most favorable pose in complex with the fibril receptor was used as the starting structure for MD. The fibril structure without a bound biflavonoid was also used to initiate an MD simulation for a control group. In an effort to compare docking scores to ThT assay EC_50_ values, a more exhaustive docking method was used. Each biflavonoid was docked using ten random seeds, and ten docking poses were generated per random seed, resulting in 100 docking poses for each biflavonoid. The generated poses were then clustered using a 2.0 Å cutoff, and the average docking score was computed for each cluster.

### 3.3. MD with Amber

The ff14SB force field [48] was applied to the protein. The tleap module of AmberTools (San Francisco, CA, USA) [49] was used to neutralize the system through the addition of Na^+^, Cu^2+^, and Cl^−^ counter ions. The system was solvated using a truncated octahedral unit cell with TIP3P water molecules [50] and a 12.0 Å solvent buffer between the solute and edge of the unit cell. GAFF forcefield parameters [51] were generated for each biflavonoid using the antechamber module of AmberTools. Constant volume and pressure were maintained during all simulations. Minimization, heating, and equilibration for each system were performed in accordance with other published works [52,53] and unrestrained MD was run at 310.0 K to a completion time of 1.0 μs for each system. This was performed with a 2.0-fs time step saving energies, coordinates, and velocities every 50,000 steps. Each of the six simulations were propagated for 1.0 μs for an overall total of 6.0 μs.

### 3.4. MD Analysis

The cpptraj module [54] of Amber16 (San Francisco, CA, USA) was used to perform root mean square deviation (RMSD), hydrogen bonding, secondary structure calculations, and principal component analysis (PCA). RMSD was used to determine the amount to which each system varied from the starting coordinates. Hydrogen bonding calculations were used to determine specific hydrogen bonds that were important for biflavonoid-fibril interaction. Hydrogen bonds were defined as having a bonding angle of 135° and a distance of 3.0 Å from acceptor to donor heavy atom. To quantify the effect of biflavonoid influence on fibril structure, per residue secondary structure was measured over the course of each trajectory by assigning each residue a DSSP value for every frame. PCA was utilized to reduce the dimensionality of the MD simulations to gain insight into biologically relevant protein motions. PCA of the coordinate covariance matrix was performed to determine the dominant motions of the peptide backbone throughout the simulated trajectories. The Normal Mode Wizard plug-in of Visual Molecular Dynamics (VMD) (Champaign, IL, USA) [55], was used to generate porcupine plots, providing a visual representation of each principal component. In order to analyze the solvation free energies of each residue for the individual systems, a per-residue free energy decomposition of each system was computed using the Molecular Mechanics/Generalized Born Surface Area (MM/GBSA) approach utilizing MMPBSA.py [56]. Per-residue free energy calculations were performed on all coordinates of the 1.0 μs simulations (60,000 frames) using the HCT Generalized Born solvation model [57]. Visualization of the completed trajectories was carried out using Visual Molecular Dynamics (VMD) [55] and UCSF Chimera (San Francisco, CA, USA) [58].

## 4. Conclusions

Aβ_42_ fibril aggregates are theorized to be toxic protein species that are pathologically linked to neurodegeneration and AD. This makes the development and discovery of aggregation inhibitors essential for AD treatment. The naturally occurring class of polyphenolic molecules known as biflavonoids exhibit activity that promotes the disaggregation of existing Aβ_42_ in vitro. Thioflavin T fluorescence assays confirm that biflavonoid compounds do exhibit these effects, and the biflavonoids differ in disaggregating activity based on structural positioning of functional groups. We utilized computational techniques to provide the first atomic details of the dynamic interactions that influence the inhibitory effects of these biflavonoids. Molecular docking calculations revealed that the biflavonoids preferentially bind to the N-terminal pocket of the Aβ_42_ fibril due to the large number of aromatic residues present in the pocket. Furthermore, MD simulations, per-residue free energy decomposition, PCA, secondary structure analysis, and hydrogen bond analysis led to several findings that help explain the inhibitory effects of the biflavonoids. Per-residue free energy decomposition calculations revealed that the biflavonoid molecules form stabilizing π–π interactions with the aromatic residues in the N-terminal pocket, which leads to a decrease in interactions between the Aβ_42_ peptides and promoting fibril destabilization. This was confirmed through secondary structure analysis which showed a larger decrease in β-sheet content over the course of the ligand-bound simulations relative to the ligand-free simulation. Additionally, PCA revealed that the biflavonoids cause the fibril structure to fold and twist, which decreases the hydrogen bonding potential within the peptide backbone of the fibril structure. Hydrogen bond analysis was further employed to elucidate the hydrogen bonding interactions between the biflavonoid and the fibril. It was determined that when hydroxyl groups are present at R_2_/R_3_, these R-groups hydrogen bond to the peptide backbone which disrupts the β-sheets of the fibril. These interactions ultimately lead to disruption of the fibril structure and suggest that hydroxyl groups at R_2_/R_3_ are key to promoting fibril deformation. Altogether, these results suggest that biflavonoids are initially bound to the Aβ_42_ fibril by the significant number of aromatic amino acids found in residues 4–14, forming favorable π–π interactions between the peptides and the biflavonoids. Once bound to this region of the fibril, the biflavonoids position themselves between the Aβ_42_ peptides and utilize the hydrogen bond donors/acceptors at the R_2_/R_3_ positions to specifically disrupt the backbone hydrogen bonding occurring between peptides in the native fibril. Our atomistic docking, simulations, and analysis support this mechanism of disaggregation for these bioflavonoids. The biflavonoid compounds presented here represent a class of molecules that can effectively act as aggregation inhibitors due to their ability to disrupt the fibril structure. Future studies will investigate biflavonoid modifications and derivatives to further examine the efficacy of these compounds as aggregation inhibitors.

Our study has several limitations. First, our experiments with the ThT assay were performed in a cell-free system; therefore, the effects of biflavonoids on disaggregation of Aβ_42_ may not be generalized to the in vitro and in vivo settings. Second, our study focused solely on the structure-activity relationship of biflavonoids in disaggregating Aβ_42_ fibrils and computational analysis of their potential interactions at the atomic levels utilizing the fibrillar structure of Aβ_42_ tetramer (PDB: 2NAO). Since amyloid plaques found in patients with AD consist of multiple Aβ species (such as Aβ_40_ and Aβ_42_) and other proteins, it is possible that biflavonoids may have different effects on disaggregating these amyloid plaques in in vivo rodent models and/or human patients with AD. Third, our data clearly demonstrated that biflavonoids directly interact with Aβ_42_ fibrils, leading to disaggregation to less ordered Aβ_42._ However, the significance of biflavonoid-driven structural changes in Aβ_42_ fibrils regarding amyloid metabolism, clearance, deposition, and neurotoxicity in cell culture as well as in vivo models remains to be determined. To address these limitations, we are currently testing how biflavonoids, in particular amentoflavone, affect the cellular clearance of Aβ aggregates in the brain parenchymal cells and the cerebrovascular cells where the extracellular deposition of Aβ aggregates occur in the brain. In addition, we are performing an in vivo study to investigate the effects of amentoflavone on Aβ disaggregation and neurological outcome using the transgenic mouse model of Alzheimer’s disease.

## Figures and Tables

**Table 1:** ΔG_decomp_, ΔG_vdw_, ΔG_ele_, ΔG_polar_, and ΔG_nonpolar_ of primary binding residues averaged across the five ligand-bound simulations.

Residue	Avg. ΔG_decomp_ (kcal·mol^−1^)	Avg. ΔG_vdw_ (kcal·mol^−1^)	Avg. ΔG_ele_ (kcal·mol^−1^)	Avg. ΔG_polar_ (kcal·mol^−1^)	Avg. ΔG_nonpolar_ (kcal·mol^−1^)
F4	−0.70 ± 1.00	−0.95 ± 1.23	−0.07 ± 0.17	+0.42 ± 0.52	−0.10 ± 0.14
H6	−0.84 ± 0.79	−1.28 ± 1.29	−0.34 ± 0.68	+0.89 ± 0.90	−0.11 ± 0.15
Y10	−1.17 ± 1.13	−1.49 ± 1.43	−0.41 ± 0.74	+0.89 ± 0.89	−0.15 ± 0.14
V12	−0.42 ± 0.65	−0.36 ± 0.34	−0.34 ± 1.24	+0.32 ± 0.71	−0.04 ± 0.05
H13	−0.48 ± 0.64	−0.74 ± 0.92	−0.24 ± 0.22	+0.57 ± 0.56	−0.07 ± 0.09
H14	−0.32 ± 0.41	−0.58 ± 0.63	−0.22 ± 0.40	+0.54 ± 0.60	−0.06 ± 0.07

**Table 2:** Percent trajectory for which R_2_ and R_3_ formed hydrogen bonds with the peptide backbone.

Compound	R_2_ (%)	R_3_ (%)
AMF	37	26
SQF	26	24
BIL	0	49
STF	75	0
PCF	11	39

## Data Availability

Not applicable.

## Supplementary Materials

The following are available online at https://www.mdpi.com/1422-0067/22/6/2888/s1. Figure S1. Projection of the individual trajectories onto modes 1 and 2 (A), modes 1 and 3 (B), and modes 2 and 3 (C) for the five ligand-bound simulations and the ligand-free simulation showing the difference in motion between systems. Figure S2. Porcupine plots of the first dominant mode of motion (A and D), second dominant mode of motion (B,E), and third dominant mode of motion (C and F) for the concatenated trajectory of the five ligand-bound simulations and the ligand-free simulation. Figure S3. (A) Change in % β-sheet content over time for the MD trajectories averaged every 25 ns. Figure S4. Decrease in β-sheet content relative to starting β-sheet content at time points: 5 ns, 50 ns, and 500 ns. Figure S5. Protein backbone hydrogen bonding analysis derived from simulation of Aβ_42_ with no ligand. Figure S6. Protein backbone hydrogen bonding analysis derived from simulation of Aβ_42_ with amentoflavone. Figure S7. Protein backbone hydrogen bonding analysis derived from simulation of Aβ_42_ with bilobetin. Figure S8. Protein backbone hydrogen bonding analysis derived from simulation of Aβ_42_ with podocarpusflavone. Figure S9. Protein backbone hydrogen bonding analysis derived from simulation of Aβ_42_ with sequoiaflavone. Figure S10. Protein backbone hydrogen bonding analysis derived from simulation of Aβ_42_ with sotetsuflavone. Table S1. ΔG_decomp_, ΔG_vdw_, ΔG_ele_, ΔG_polar_, and ΔG_nonpolar_ of all residues averaged across the five ligand-bound simulations. Table S2. Total number of backbone hydrogen bonds on the Aβ_42_ fibril and their associated prevalence during each 1.0-μs simulation performed.
