# Supplementary material for: Biflavonoid-Induced Disruption of Hydrogen Bonds Leads to Amyloid-β Disaggregation
Source: Int J Mol Sci. 2021 Mar 12;22(6):2888. doi: 10.3390/ijms22062888 (PMC8001082; doi:10.3390/ijms22062888)
Supplement: Supplementary file 1 [file ijms-22-02888-s001.pdf]

SUPPORTING INFORMATION

Table S1. ΔG_{decomp}, ΔG_{vdw}, ΔG_{ele}, ΔG_{polar}, and $\Delta G_{nonpolar}$ of all residues averaged across the five ligand-bound simulations.

Residue	Avg. ΔG_{decomp} (kcal·mol^{-1})	Avg. ΔG_{vdw} (kcal·mol^{-1})	Avg. ΔG_{ele} (kcal·mol^{-1})	Avg. ΔG_{polar} (kcal·mol^{-1})	Avg. $\Delta G_{nonpolar}$ (kcal·mol^{-1})
D1	+0.18 ± 0.17	-0.16 ± 0.23	-0.06 ± 0.27	+0.43 ± 0.52	-0.02 ± 0.04
A2	-0.34 ± 0.60	-0.33 ± 0.66	-0.23 ± 0.55	+0.28 ± 0.56	-0.06 ± 0.11
E3	-0.11 ± 0.49	-0.22 ± 0.35	-0.49 ± 1.59	+0.63 ± 1.54	-0.03 ± 0.07
F4	-0.70 ± 1.00	-0.95 ± 1.23	-0.07 ± 0.17	+0.42 ± 0.52	-0.10 ± 0.14
R5	-0.15 ± 0.24	-0.25 ± 0.28	-0.43 ± 0.34	+0.55 ± 0.35	-0.01 ± 0.03
H6	-0.84 ± 0.79	-1.28 ± 1.29	-0.34 ± 0.68	+0.89 ± 0.90	-0.11 ± 0.15
D7	-0.11 ± 0.37	-0.24 ± 0.49	-0.16 ± 1.24	+0.32 ± 1.09	-0.03 ± 0.06
S8	-0.20 ± 0.26	-0.31 ± 0.27	-0.30 ± 0.57	+0.44 ± 0.64	-0.03 ± 0.04
G9	-0.27 ± 0.30	-0.36 ± 0.41	-0.38 ± 0.84	+0.51 ± 0.60	-0.03 ± 0.03
Y10	-1.17 ± 1.13	-1.49 ± 1.43	-0.41 ± 0.74	+0.89 ± 0.89	-0.15 ± 0.14
E11	-0.23 ± 0.47	-0.23 ± 0.48	-1.37 ± 2.67	+1.41 ± 2.42	-0.04 ± 0.07
V12	-0.42 ± 0.65	-0.36 ± 0.34	-0.34 ± 1.24	+0.32 ± 0.71	-0.04 ± 0.05
H13	-0.48 ± 0.64	-0.74 ± 0.92	-0.24 ± 0.22	+0.57 ± 0.56	-0.07 ± 0.09
H14	-0.32 ± 0.41	-0.58 ± 0.63	-0.22 ± 0.40	+0.54 ± 0.60	-0.06 ± 0.07
Q15	0.00 ± 0.03	-0.04 ± 0.05	-0.02 ± 0.11	+0.07 ± 0.12	0.00 ± 0.00
K16	+0.03 ± 0.03	-0.02 ± 0.01	-0.05 ± 0.22	+0.09 ± 0.21	0.00 ± 0.00
L17	0.00 ± 0.00	0.00 ± 0.00	+0.01 ± 0.01	0.00 ± 0.01	0.00 ± 0.00
V18	0.00 ± 0.00	0.00 ± 0.00	0.00 ± 0.01	+0.01 ± 0.01	0.00 ± 0.00
F19	0.00 ± 0.00	0.00 ± 0.00	0.00 ± 0.00	0.00 ± 0.00	0.00 ± 0.00
F20	0.00 ± 0.00	0.00 ± 0.00	0.00 ± 0.00	0.00 ± 0.00	0.00 ± 0.00
A21	0.00 ± 0.00	0.00 ± 0.00	0.00 ± 0.00	0.00 ± 0.00	0.00 ± 0.00
E22	0.00 ± 0.00	0.00 ± 0.00	-0.02 ± 0.08	+0.02 ± 0.08	0.00 ± 0.00
D23	0.00 ± 0.00	0.00 ± 0.00	-0.01 ± 0.06	+0.01 ± 0.06	0.00 ± 0.00
V24	0.00 ± 0.00	0.00 ± 0.00	0.00 ± 0.00	0.00 ± 0.00	0.00 ± 0.00
G25	0.00 ± 0.00	0.00 ± 0.00	0.00 ± 0.00	0.00 ± 0.00	0.00 ± 0.00
S26	0.00 ± 0.00	0.00 ± 0.00	0.00 ± 0.00	0.00 ± 0.00	0.00 ± 0.00
N27	0.00 ± 0.00	0.00 ± 0.00	0.00 ± 0.00	0.00 ± 0.00	0.00 ± 0.00
K28	0.00 ± 0.00	0.00 ± 0.00	-0.01 ± 0.09	+0.01 ± 0.09	0.00 ± 0.00
G29	0.00 ± 0.00	0.00 ± 0.00	0.00 ± 0.00	0.00 ± 0.00	0.00 ± 0.00
A30	0.00 ± 0.00	0.00 ± 0.00	0.00 ± 0.00	0.00 ± 0.00	0.00 ± 0.00
I31	0.00 ± 0.00	0.00 ± 0.00	0.00 ± 0.00	0.00 ± 0.00	0.00 ± 0.00
I32	0.00 ± 0.00	0.00 ± 0.00	0.00 ± 0.00	0.00 ± 0.00	0.00 ± 0.00
G33	0.00 ± 0.00	0.00 ± 0.00	0.00 ± 0.00	0.00 ± 0.00	0.00 ± 0.00
L34	0.00 ± 0.00	0.00 ± 0.00	0.00 ± 0.03	0.00 ± 0.01	0.00 ± 0.00
M35	0.00 ± 0.00	0.00 ± 0.00	0.00 ± 0.00	0.00 ± 0.00	0.00 ± 0.00
V36	0.00 ± 0.00	0.00 ± 0.00	0.00 ± 0.00	0.00 ± 0.00	0.00 ± 0.00

G37	0.00 ± 0.00	0.00 ± 0.00	0.00 ± 0.00	0.00 ± 0.00	0.00 ± 0.00
G38	0.00 ± 0.00	0.00 ± 0.00	0.00 ± 0.00	0.00 ± 0.00	0.00 ± 0.00
V39	0.00 ± 0.00	0.00 ± 0.00	0.00 ± 0.00	0.00 ± 0.00	0.00 ± 0.00
V40	0.00 ± 0.00	0.00 ± 0.00	0.00 ± 0.00	0.00 ± 0.00	0.00 ± 0.00
I41	0.00 ± 0.00	0.00 ± 0.00	0.00 ± 0.00	0.00 ± 0.00	0.00 ± 0.00
A42	0.00 ± 0.00	0.00 ± 0.00	0.00 ± 0.03	0.00 ± 0.03	0.00 ± 0.00

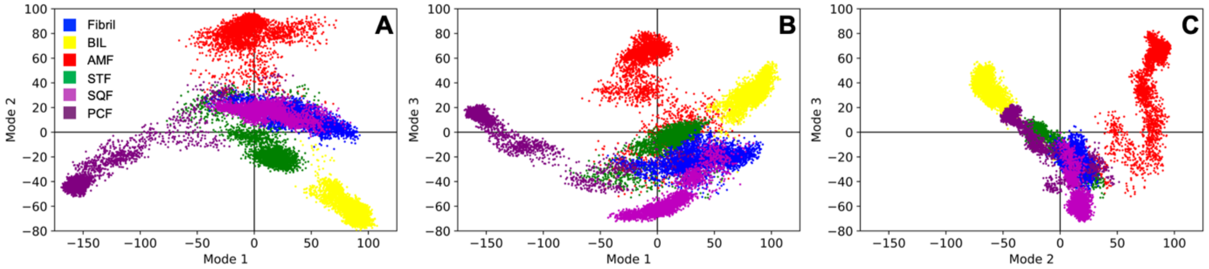

Figure S1. Projection of the individual trajectories onto modes 1 and 2 (A), modes 1 and 3 (B), and modes 2 and 3 (C) for the five ligand-bound simulations and the ligand-free simulation showing the difference in motion between systems.

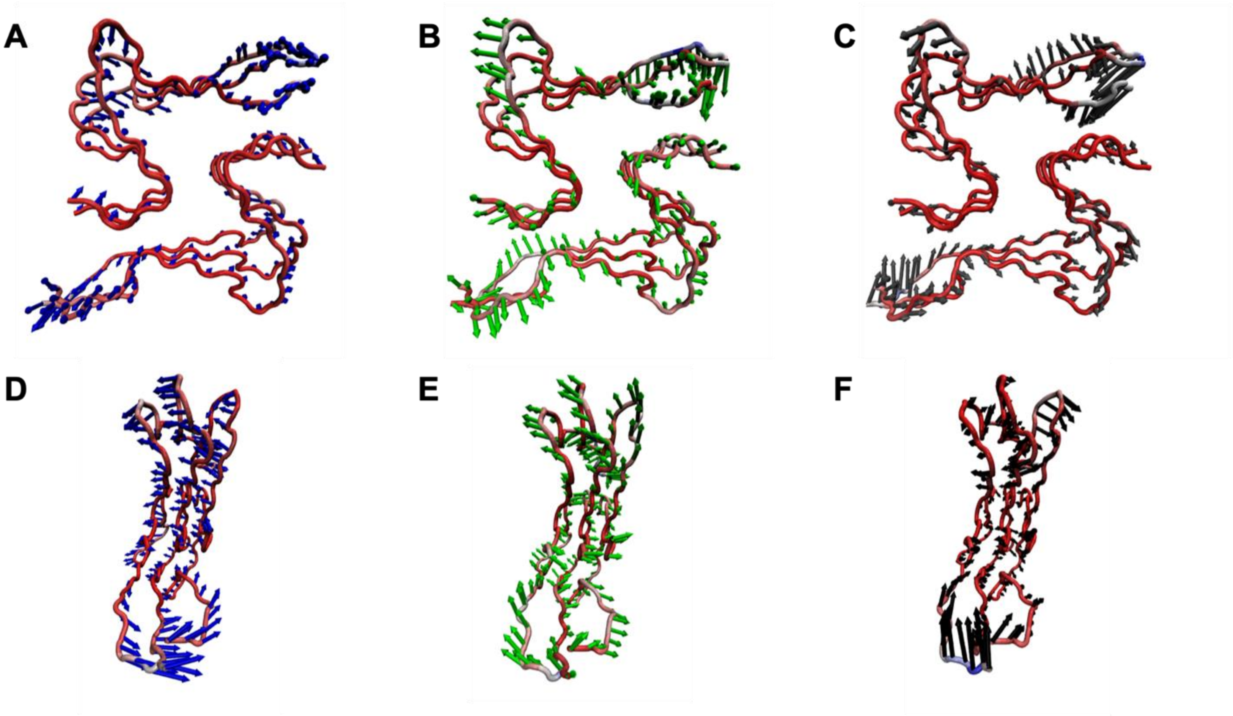

Figure S2. Porcupine plots of the first dominant mode of motion (A and D), second dominant mode of motion (B and E), and third dominant mode of motion (C and F) for the concatenated trajectory of the five ligand-bound simulations and the ligand-free simulation. Vectors represent general motion of each α-carbon in the fibril.

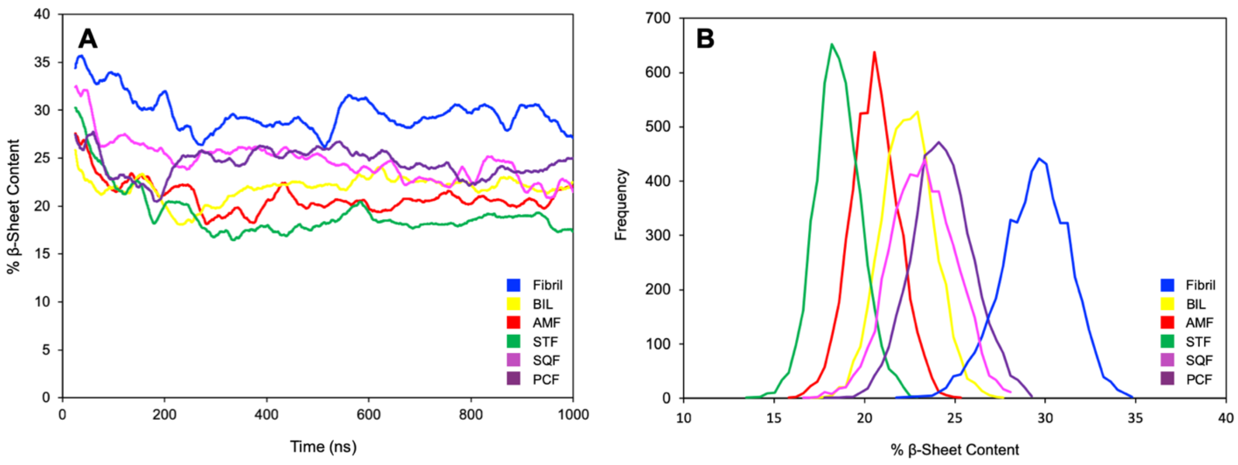

Figure S3. (A) Change in % β-sheet content over time for the MD trajectories averaged every 25 ns. Ligand-bound simulations shows greater β-sheet content relative to the ligand-free simulation. Note that all simulations started at the same % β-sheet content (~35%), but due to the moving average calculation, the first 25 ns were not able to be captured graphically resulting in varying starting points. (B) Histogram showing the β-sheet conformations sampled over the last 500 ns of the MD trajectories. Ligand-free simulation shows greater β-sheet content relative to the ligand-bound simulations.

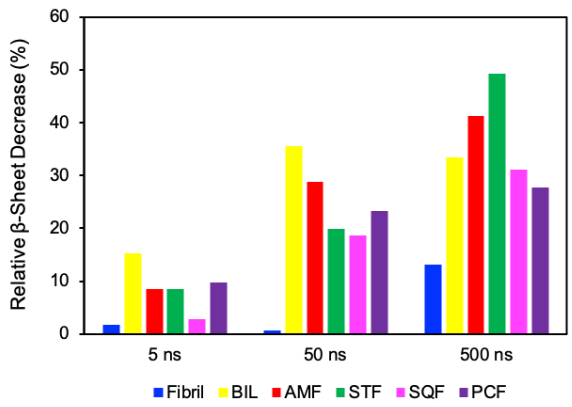

Figure S4. Decrease in β-sheet content relative to starting β-sheet content at time points: 5 ns, 50 ns, and 500 ns.

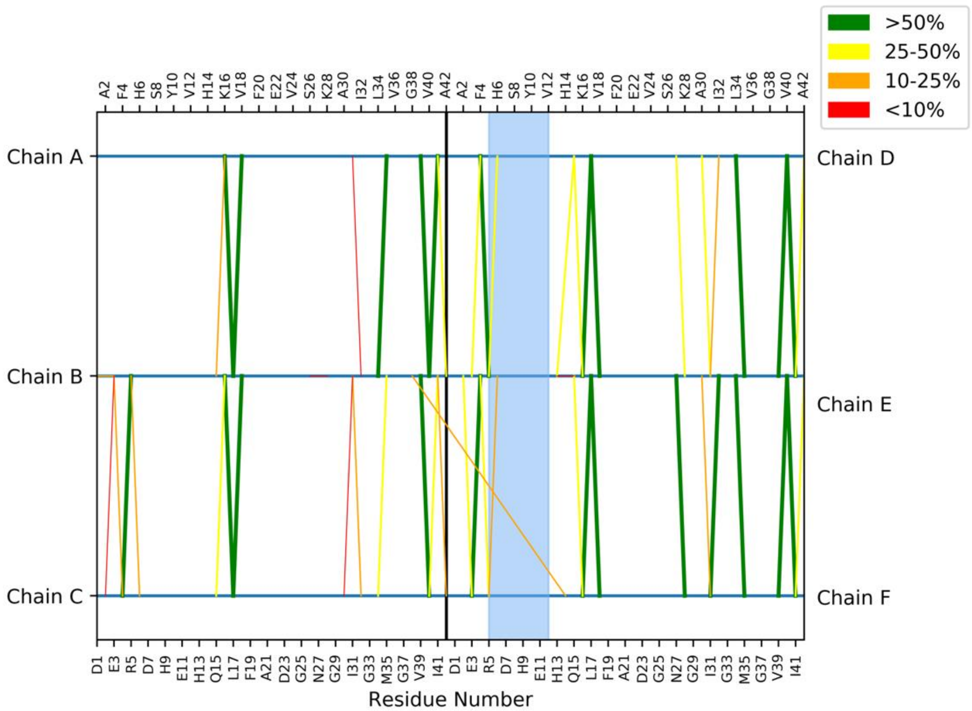

Figure S5. Protein backbone hydrogen bonding analysis derived from simulation of Aβ_{42} with no ligand. Hydrogen bonds percentages were calculated over the 1.0-μs simulation, and were categorized by the percent of frames with backbone hydrogen bonds present: >50% (green), 25-50% (yellow), 10-25% (orange) and less than 10% (red). Each line depicted shows a backbone hydrogen bond present during the simulation to and from one of the six peptides (Chains A-F) from the original structure (PDB 2NAO). The blue highlighted region depicts the area with the most direct contact with each ligand from docking.

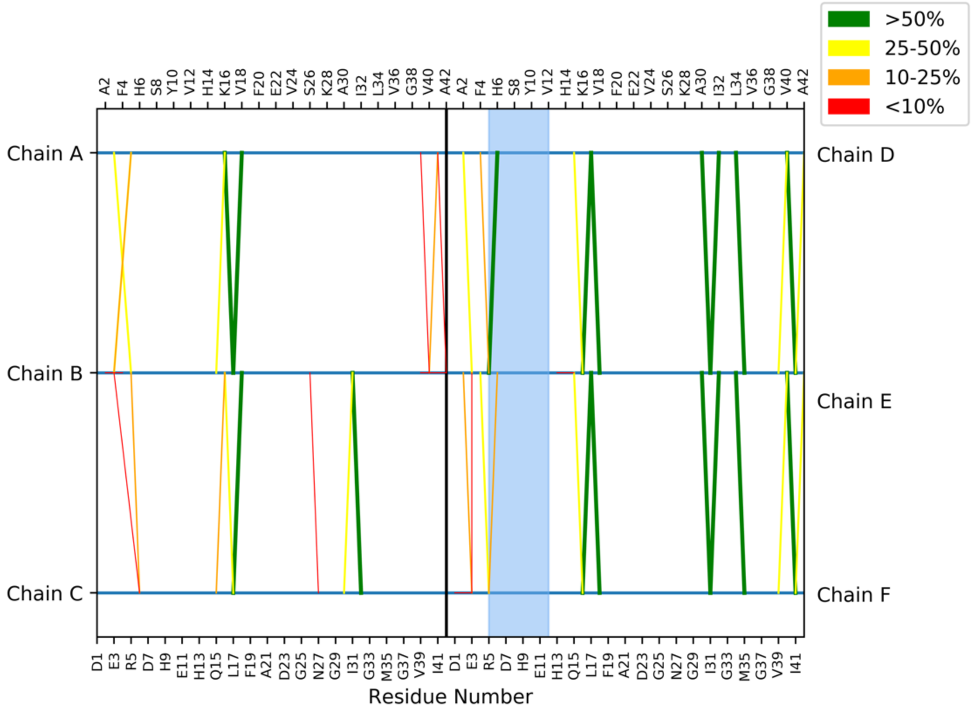

Figure S6. Protein backbone hydrogen bonding analysis derived from simulation of Aβ_{42} with amentoflavone. Hydrogen bonds percentages were calculated over the 1.0-μs simulation, and were categorized by the percent of frames with backbone hydrogen bonds present: >50% (green), 25-50% (yellow), 10-25% (orange) and less than 10% (red). Each line depicted shows a backbone hydrogen bond present during the simulation to and from one of the six peptides (Chains A-F) from the original structure (PDB 2NAO). The blue highlighted region depicts the area with the most direct contact with each ligand from docking.

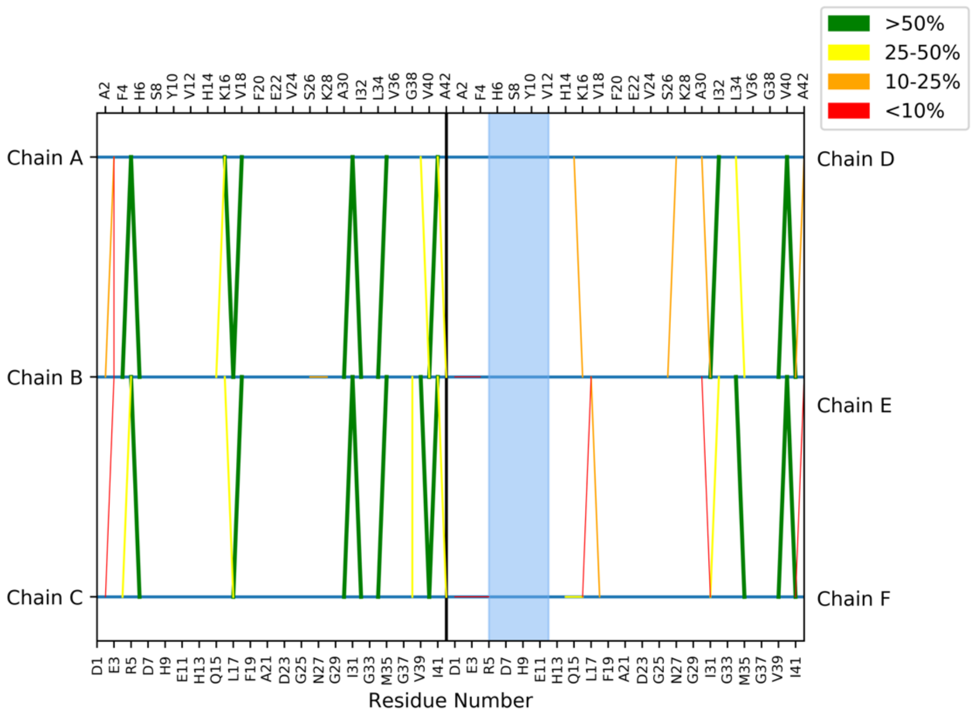

Figure S7. Protein backbone hydrogen bonding analysis derived from simulation of Aβ_{42} with bilobetin. Hydrogen bonds percentages were calculated over the 1.0-μs simulation, and were categorized by the percent of frames with backbone hydrogen bonds present: >50% (green), 25-50% (yellow), 10-25% (orange) and less than 10% (red). Each line depicted shows a backbone hydrogen bond present during the simulation to and from one of the six peptides (Chains A-F) from the original structure (PDB 2NAO). The blue highlighted region depicts the area with the most direct contact with each ligand from docking.

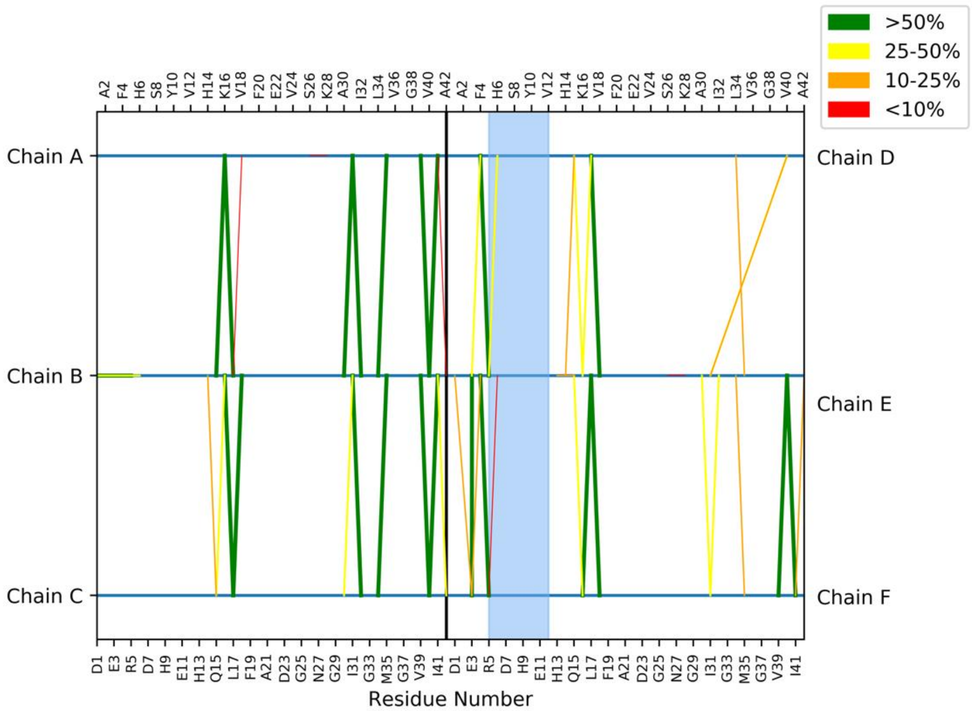

Figure S8. Protein backbone hydrogen bonding analysis derived from simulation of Aβ_{42} with podocarpusflavone. Hydrogen bonds percentages were calculated over the 1.0-μs simulation, and were categorized by the percent of frames with backbone hydrogen bonds present: >50% (green), 25-50% (yellow), 10-25% (orange) and less than 10% (red). Each line depicted shows a backbone hydrogen bond present during the simulation to and from one of the six peptides (Chains A-F) from the original structure (PDB 2NAO). The blue highlighted region depicts the area with the most direct contact with each ligand from docking.

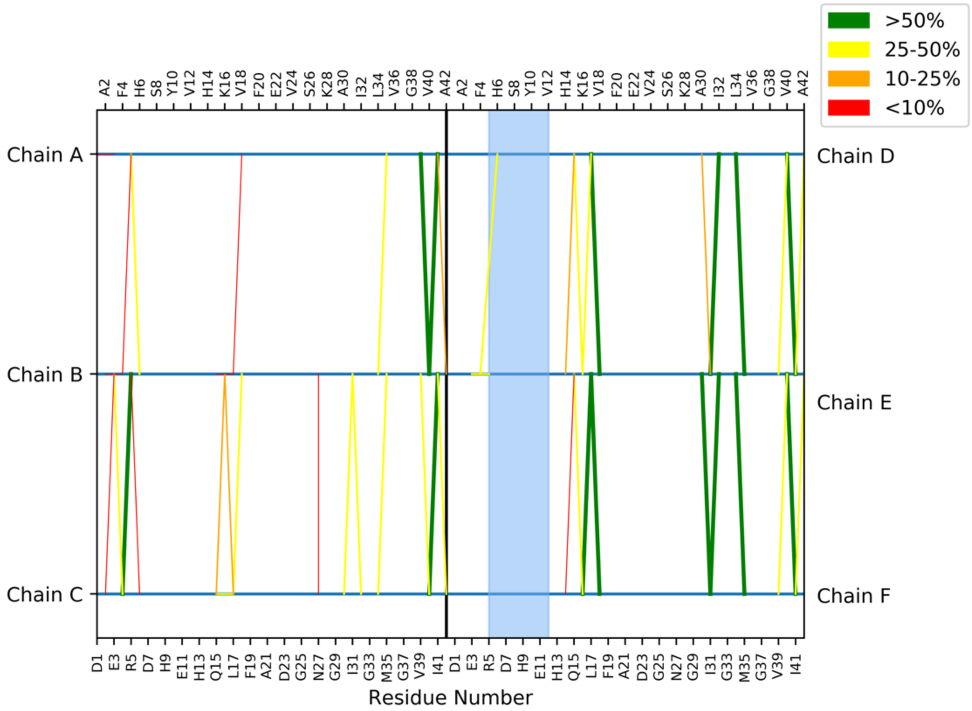

Figure S9. Protein backbone hydrogen bonding analysis derived from simulation of Aβ_{42} with sequoiaflavone. Hydrogen bonds percentages were calculated over the 1.0-μs simulation, and were categorized by the percent of frames with backbone hydrogen bonds present: >50% (green), 25-50% (yellow), 10-25% (orange) and less than 10% (red). Each line depicted shows a backbone hydrogen bond present during the simulation to and from one of the six peptides (Chains A-F) from the original structure (PDB 2NAO). The blue highlighted region depicts the area with the most direct contact with each ligand from docking.

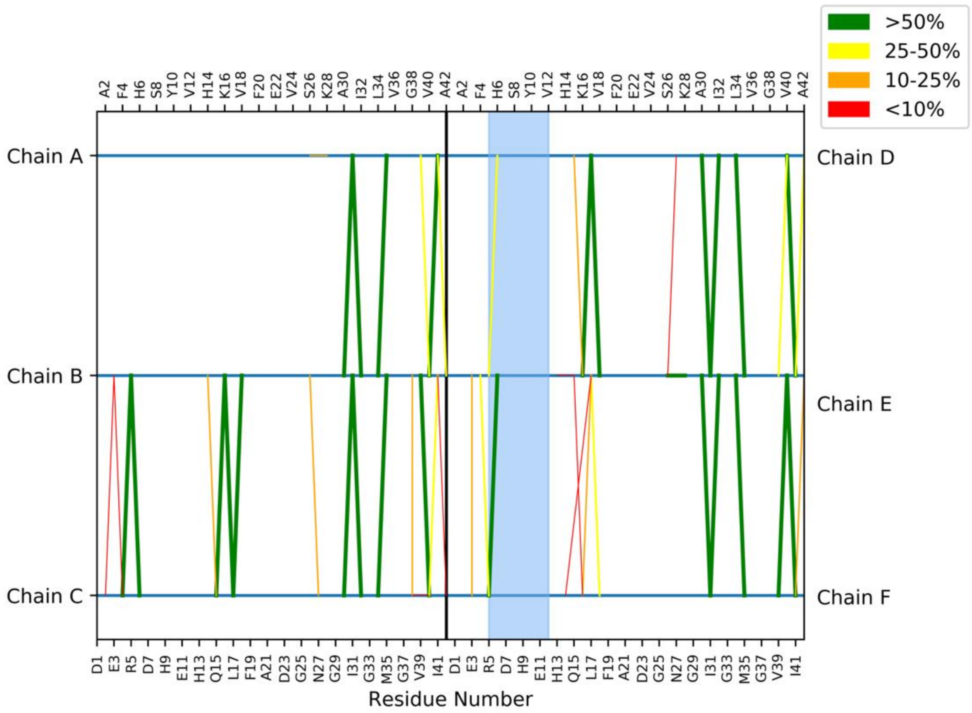

Figure S10. Protein backbone hydrogen bonding analysis derived from simulation of Aβ_{42} with sotetsuflavone. Hydrogen bonds percentages were calculated over the 1.0-μs simulation, and were categorized by the percent of frames with backbone hydrogen bonds present: >50% (green), 25-50% (yellow), 10-25% (orange) and less than 10% (red). Each line depicted shows a backbone hydrogen bond present during the simulation to and from one of the six peptides (Chains A-F) from the original structure (PDB 2NAO). The blue highlighted region depicts the area with the most direct contact with each ligand from docking.

Table S2. Total number of backbone hydrogen bonds on the Aβ_{42} fibril and their associated prevalence during each 1.0-μs simulation performed.

	Number of backbone hydrogen bonds				
**Simulation**	**>50%**	**25-50%**	**10-25%**	**<10%**	**Total H-Bonds**
Ligand-free	23	15	10	5	53
AMF	17	14	7	9	47
BIL	21	10	7	7	45
PCF	22	13	9	5	49
SQF	14	19	5	9	47
STF	26	8	8	8	50
